# Evolution of High Trophic Diversity Based on Limited Functional Disparity in the Feeding Apparatus of Marine Angelfishes (f. Pomacanthidae)

**DOI:** 10.1371/journal.pone.0024113

**Published:** 2011-09-01

**Authors:** Nicolai Konow, David R. Bellwood

**Affiliations:** School of Marine and Tropical Biology, and Australian Research Council Centre of Excellence for Coral Reef Studies, James Cook University, Townsville, Australia; Swansea University, United Kingdom

## Abstract

The use of biting to obtain food items attached to the substratum is an ecologically widespread and important mode of feeding among aquatic vertebrates, which rarely has been studied. We did the first evolutionary analyses of morphology and motion kinematics of the feeding apparatus in Indo-Pacific members of an iconic family of biters, the marine angelfishes (f. Pomacanthidae). We found clear interspecific differences in gut morphology that clearly reflected a wide range of trophic niches. In contrast, feeding apparatus morphology appeared to be conserved. A few unusual structural innovations enabled angelfishes to protrude their jaws, close them in the protruded state, and tear food items from the substratum at a high velocity. Only one clade, the speciose pygmy angelfishes, showed functional departure from the generalized and clade-defining grab-and-tearing feeding pattern. By comparing the feeding kinematics of angelfishes with wrasses and parrotfishes (f. Labridae) we showed that grab-and-tearing is based on low kinematics disparity. Regardless of its restricted disparity, the grab-and-tearing feeding apparatus has enabled angelfishes to negotiate ecological thresholds: Given their widely different body sizes, angelfishes can access many structurally complex benthic surfaces that other biters likely are unable to exploit. From these surfaces, angelfishes can dislodge sturdy food items from their tough attachments. Angelfishes thus provide an intriguing example of a successful group that appears to have evolved considerable trophic diversity based on an unusual yet conserved feeding apparatus configuration that is characterized by limited functional disparity.

## Introduction

Structural and functional attributes of the feeding apparatus are considered important features in promoting the impressive evolutionary diversification and ecological success of teleosts, or modern bony fishes [1]. Ecomorphological analyses, aiming at identifying relationships between form, function and ecology, have typically focused on marine wrasses and rift-lake cichlids that use ram-feeding to overtake and engulf prey, or suction feeding to draw prey into the mouth [2], [3], [4]. Most ecomorphological analyse have examined structural diversity and modelled the biomechanics of jaw movement used in ram and suction feeding in the water column. Meanwhile, feeding apparatus motion-patterns (kinematics) have rarely been evaluated, although this functional component is argued as key in identifying ecomorphological relationships [5].

Data on how the feeding apparatus is configured, but not on how it actually moves, has led to the concept of ‘many-to-one mapping’, which explains the relationship between structural redundancy and functional convergence [6], [7]. Given the taxonomic study-emphasis outlined above, free-water feeding taxa have become the exemplars of many-to-one mapping [8], [9], [10]. Therefore, it now becomes important to examine the generality of the many-to-one mapping concept. We initiate this aim by studying feeding kinematics sampled systematically from multiple species across a speciose and predominately biting clade.

The importance of ecomorphological quantification of biting has often been noted [5], [11], [12], [13] but rarely carried out, perhaps owing to the general intractability of biters in captivity. As a consequence, few data exist on feeding apparatus kinematics in biting taxa that forage on physically heterogeneous aquatic substrata (but see [14]). This is unfortunate, not only because biting likely is the most derived of fish feeding modes [11], [15], but especially because biters are exceptionally widespread in high-diversity ecosystems, including African rift lakes and tropical marine rocky and coral reefs [1], [16]. Moreover, biters are often identified as key to the preservation and maintenance of ecosystem resilience [17], [18], [19]. Paradoxically, while biters are evolutionarily interesting and ecologically important, they remain among the taxa that are the least understood from a functional perspective.

Marine angelfishes (f. Pomacanthidae) are especially worthy of detailed analyses to redress this research imbalance. Historically, it is only their morphology that has been examined [20], [21], [22], [23]. However, a single analysis did identify functional novelties in the feeding apparatus of a generalized species [24]. These novelties include rotation of the suspensorium (cheek region), which enables the lower jaw to protrude forward. This is an extraordinarily rare trait among bony fishes [25]. Moreover, closure of the protruded mouth onto the food item is enabled by an extra intramandibular joint in the lower jaw. After grabbing the food item, the closed jaws are retracted at a high velocity, to tear the food item from its attachment site. In combination, these novel functional traits yield a previously unrecognized ‘grab-and-tearing’ feeding mode, useful for severing the sturdy attachment of tough-bodied benthic invertebrates [24]. However, a single-taxon analysis cannot reveal the evolutionary development of grab-and-tearing, nor can it quantify the role that key innovations have played in the evolution of angelfish structural and functional disparity.

Field observations suggest that angelfishes occupy a diverse range of trophic niches, ranging from spongivory and herbivory to planktivory [26], [27], [28]. Herbivory has been linked with hindgut fermentation in some taxa [29], [30], but gut morphology has otherwise not been used to delineate the angelfish trophic niches, a method that has been effective in studies of several other groups [31].

Here, we study feeding apparatus structure and function among Indo-Pacific marine angelfishes. A phylogeny for the family permits us to attain a balanced analysis of interspecific variation in feeding apparatus form and function across a predominantly biting marine fish lineage. First, we evaluate the level of structural variation in the angelfish feeding apparatus, compared with earlier studies [20], [21], [22], [23], [24]. Then, we examine gut morphology to evaluate the previously proposed range of trophic niches. Motion analyses of feeding in eight Indo-West Pacific species, representing all major lineages, are then used to quantify the diversity in biting kinematics at the family-level. Finally, to quantify the disparity of feeding apparatus function, we compare angelfish biting kinematics with similar data from the well studied wrasses and parrotfishes (f. Labridae).

Given that angelfishes appear to occupy a wide range of ecological niches, we hypothesise that their feeding apparatus is characterized by a high level of structural diversity. Moreover, we hypothesize that this structural diversity is reflected by clear differences in biting kinematics across the family. Finally, we hypothesise that feeding kinematics are correlated with, and mechanically linked with, variation in trophic niches, with the alternative being many-to-one mapping between feeding morphology and kinematics.

## Materials and Methods

### Ethics statement

All results reported in this paper were generated via research endorsed by a Great Barrier Reef Marine Park collection and research permit (G01/257_1) and by a James Cook University Ethics Approval (A657/01).

### Selection and collection of study taxa

Taxon selection followed a phylogeny derived from 12S and 16S mDNA [32], with the specific study taxa (Table 1) chosen so as to obtain an even representation in the analysis of all lineages occurring on the Great Barrier Reef (GBR), Australia. The GBR angelfish assemblage includes representatives from 10 of the 12 recognized genera (83%). Species-selection was directed towards the most abundant and widespread taxa. For each of the eight primary study species, a minimum of 3 specimens were collected on SCUBA from the central and northern GBR using barrier nets, or hand nets and clove oil. The adult body size of angelfishes ranges across an order of magnitude [27], [28]. In order to reduce scaling-effects in our kinematics data [33] we obtained specimens of smaller taxa at their maximum body size (Table 1).

**Table 1:** Summary of taxa examined. Listed according to phylogenetic ranking (Figure 1).

Genus	Subgenus	Species	Code	TL [mm] min-max (mean)	HL [mm] min-max (mean)	R.G.I. Mean (S.E.M.)	Total Examined	Kinematics	Dissected & Clear-stained
*Centropyge*	*Centropyge*	*Bicolour*	Cc	115-109 (112)	25-22 (23)	3.3 (0.1)	6	3	3
*Apolemichthys*		*trimaculatus*	A	156-144 (151)	36-35 (37)	3.3 (0.5)	6	3	3
*Genicanthus*		*melanospilos*	G	147-113 (130)	27-22 (25)	1.3 (0.1)	6	3	3
*Centropyge*	*Xiphypops*	*Bispinosa*	Cx	110-84 (101)	23-19 (22)	10.3 (0.1)	6	3	3
*Paracentropyge*		*multifasciata*	Pc	68-60 (65)	19-18 (18)	2.9 (0.2)	3	-	3
*Pygoplites*		*diacanthus*	P	151-145 (147)	39-37 (38)	5.8 (0.2)	6	3	3
*Chaetodontoplus*		*duboulayi*	C	234-210 (220)	47-42 (44)	4.1 (0.1)	6	3	3
*Pomacanthus*	*Euxiphipops*	*sexstriatus*	Pe	313-257 (291)	66-64 (65)	7.6 (0.5)	6	3	3
*Pomacanthus*	*Arusetta*	*semicirculatus*	Pa	408-227 (295)	85-51 (68)	2.5 (0.5)	8	3	5
*Pomacanthus*	*Acanthochaetodon*	*Imperator*	Pc	204-126 (174)	47-31 (41)	4.5 (0.6)	3	-	3

The following species were sampled (listed with their trophic niche status, following [26], [27], [28], [34]): *Centropyge [Centropyge] bicolor* and *Apolemichthys trimaculatus* are both gracile omnivores that feed on zoobenthos; *Genicanthus melanospilos* is a zooplanktivore that occasionally bites attached invertebrate food items; *Centropyge [Xiphypops] bispinosa* is a herbivore; *Pygoplites diacanthus* is an omnivore that feeds on attached invertebrates; *Chaetodontoplus duboulayi* is an omnivore that feeds on sponges and tunicates; *Pomacanthus [Euxiphipops] sexstriatus* is a herbivore that feeds on calcareous and turf algae; *Pomacanthus [Arusetta] semicirculatus* is a carnivore that feeds on sturdy invertebrate food items (e.g. poriferan sponges and tunicates).

We follow the taxonomy of [32], who rejected the subgenus *Pomacanthus [Pomacanthodes]*, leading us to provisionally adopt suggestions from [35] regarding the sub-generic classification of *Pomacanthus* (for alternative views, see [36]). The species *Paracentropyge multifasciata* was too shy for video filming, and *Pomacanthus imperator*, the sole GBR representative of *Pomacanthus [Acanthochaetodon]* Bleeker 1876 could not be obtained live for filming. However, the latter taxon is morphologically very similar to and shares a similar trophic ecology with *Pomacanthus [Arusetta] semicirculatus* [27].

### Husbandry and experimental design

**Figure 1:**
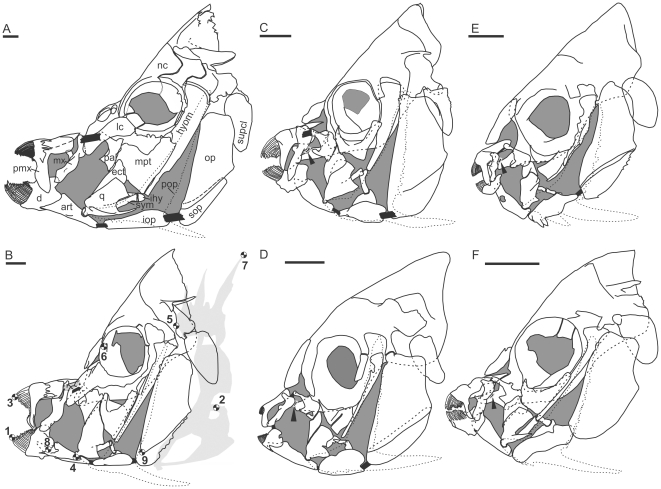
Restricted structural diversity in the angelfish feeding apparatus drawn from clear-stained and dissected specimens (**Table 1**). A, *Pomacanthus [Arusetta] semicirculatus*; B, *Chaetodontoplus duboulayi*; C, *Pygoplites diacanthus*; D, *Genicanthus melanospilos*; E, *Centropyge [Xiphypops] bispinosa*; F, *Centropyge [Centropyge] bicolor*. Bone labelling in A (B–F follows): art, articular; d, dentary; ect, ectopterygoid; hyom, hyomandibular; ihy, interhyal; iop, interoperculum; mpt, metapterygoid; mx, maxilla; nc, neurocranium; op, operculum; pal, palatine; pmx, premaxilla; pop, preoperculum (in fine stippling); q, quadrate; sop, suboperculum; supcl, supracleitrum; sym, symplectic; lc, lachrymal; v, vomer. Shading denotes space not occupied by bone; ligaments are shown in black. Black shapes with white margin, as indicated with black arrowheads in C–F, are cartilaginous discs unique to the pygmy angelfish clade (Figure 2). Note the reduced suspensoria in *Genicanthus* (C) and *C. [Xiphypops]* (D). For clarity, drawings only include cranial structures, except in (B), where the positions of reflective markers glued to the skin of kinematics study animals are indicated: 1, tip of anterior-most lower jaw; 2, base of pectoral fin; 3, anterior-most upper jaw; 4, mandibular (ancestral) lower jaw joint; 5, posterior joint between cheek region and (suspensorium) and neurocranium; 6, reference point in front of eye; 7, base of first dorsal spine; 8, intramandibular (derived) lower jaw joint; 9, ligamentous link between bones of the gill cover. Scale bar, 5 mm.

Specimens were individually housed in aquaria, where they were maintained and filmed following protocols detailed earlier [24]. Animals were encouraged to feed in a narrow passage between the aquarium front glass and a reference-grid background. During acclimation, specimens were trained to feed under floodlight illumination on food items that were fixed in place using a spring-loaded stainless steel crocodile clip firmly mounted on the floor of the feeding passage. During feeding trials, rock oyster shells of uniform size (5–6 cm^2^ surface area) covered with a mixed epifauna of sponges, turf algae, ascidians, tubeworms, and tunicates were collected from local coastal marine pylons. A major advantage of this food-treatment was that all taxa would feed on it. This minimizes the risk of introducing critical prey-type or prey-size effects into the resulting dataset. For husbandry purposes, both rock oyster shell epifauna and live ghost shrimp (*Acetes* sp.) collected in adjacent waters were provided. Prior to experiments, reflective markers were glued to the fish skin as reference markers for motion analysis. These markers were carefully attached over joints of interest in the jaws, suspensorium, pectoral girdle and the craniovertebral joint, and as reference markers at the bases of the dorsal, pectoral and pelvic fins (Figure 1B).

### Feeding performance testing and morphological sampling

To ensure a perpendicular orientation of the reflective markers to the camera lens axis, fish were presented with attached food in the feeding passage. Meanwhile, high-speed video was recorded using a JVC GR-DVL9800u digital video camera. The JVC video stream was split into 200 images s^−1^ using custom Matlab scripts and commercially available software (see details in [24]). *Genicanthus* specimens were recorded using a NAC Memrecam CI at 400 images s^−1^ in the Wainwright lab at UC Davis. No less than three feeding events for each specimen were analysed. Our use of large specimens increased the temporal resolution of kinematics in our high-speed video sequences. It also prevented ontogenetic effects on kinematics [33]. Performance-maxima were the focus of this study. Therefore, we prioritized aggressive bites, selecting only the fastest of bites that fulfilled all other analysis criteria (see below). This approach also reduced, if not excluded, the effects of satiation [37], and diminished the variability in timing and duration of the preparatory and expansive phases of bites that was reported earlier [24].

**Figure 2:**
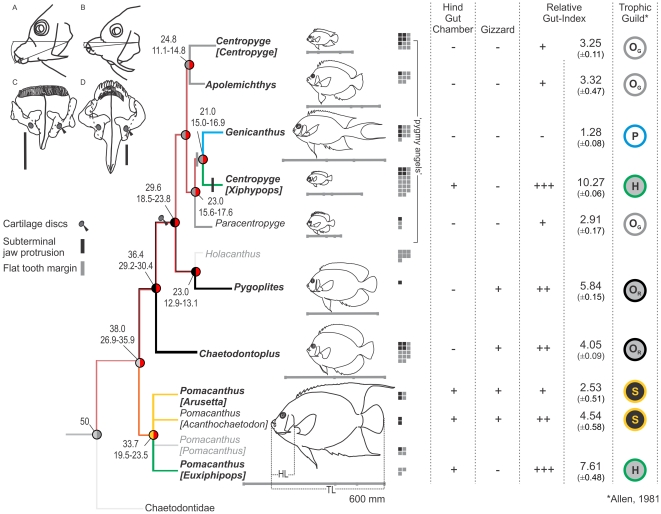
Complex evolution of angelfish ecological diversity. Tracings of video images showing different jaw protrusion patterns in (A) *Centropyge [Xiphypops]* (ventral), vs. (B) *Centropyge [Centropyge]*, and all other taxa (forward). Upper jaw structures (front facing up; scale bar, 5 mm) showing cartilage discs (black arrows) in the pygmy angelfish clade (Figure 1) and difference in jaw profile between (C) *C. [Xiphypops]* and *Genicanthus* (flat) vs. (D) *Pygoplites* and all other taxa (curved). In the character matrix, relative gut length (mean ±S.D.) is coded as a discrete variable, and presence/absence of hindgut chambers and gizzards is shown. Trophic niche predictions are indicated on the far right. Note the complete correspondence as morphological character-states are optimised to the phylogeny under squared-change parsimony (Mesquite v. 2.5): H, herbivore; O_G_, gracile omnivore; O_R_, robust omnivore; P, planktivore, S, spongivore. Herbivory evolved independently in *Pomacanthus [Euxiphipops]* and *C. [Xiphypops]*, which differ from congeners by having three times higher relative gut indices, presence of a hindgut chamber, and no gizzard. Large-bodied spongivores have short guts with a gizzard and a hindgut chamber. Robust omnivores share a medium-length gizzard-bearing gut but no hindgut. A short unspecialised gut unites the gracile omnivores. The planktivore *Genicanthus* resembles gracile omnivores but has the shortest gut in the family.

Following video recordings, specimens were euthanized with an overdose of Eugenol (Clove oil), and total length (TL), standard length (SL) and head length (HL) measurements were taken (Table 1). Specimens were then either dissected fresh or fixed in formalin for tissue-clearing and bone-cartilage counterstaining [24]. Clear-stained and dissected specimens were manipulated in order to examine the articulations of the jaws, suspensorium and hyoid with the neurocranium and pectoral girdle. Morphological diagrams were drawn directly from these preparations using a stereo microscope with a Camera Lucida attachment and digitised in Corel Draw v.12 (figs. 1, 2). Bone and soft tissue nomenclature followed [24]. Gut data were obtained from freshly killed specimens directly off the reef [31]. Viscera were excised from three unpreserved and even-sized specimens from each species and carefully disentangled to measure the extended gut from the posterior-most point of the stomach to the vent, including the hindgut chamber length, where present. Gut lengths were standardised with TL and Mean ± S.E.M of the relative gut length indices were calculated (Figure 2).

### Morphological and kinematics data analyses

The correspondence between previously inferred trophic niches [26], [27], [28], [34] and groupings of taxa based on their jaw morphological specializations (cartilage discs, flat frontal tooth margin and sub-terminal jaw protrusion), relative gut length and presence or absence of hindgut fermenting chambers and gizzards was visualised by mapping of phylogenetic traits (Figure 2) under squared-change parsimony (Mesquite v. 2.5).

Video sequences were analysed only when the entire feeding event was completed in focus and recorded in lateral profile. The total duration of feeding events (*t*_TOT_) were cropped from protrusion-onset (*t*_S_), via time of bite (*t*_B_) to completed jaw retraction (*t*_C_) using Virtual Dub (v.1.7.4). The nine reflective markers (positions shown in Figure 1B) were tracked semi-automatically in Movias Pro (v.1.5). The resulting columns of x∶y coordinates were used to calculate linear excursions (distances between coordinate pairs) and angular excursions (between coordinate pairs for three points), as well as onset-timing, duration and velocity for seven joints and linkages: gape opening and closure, lower jaw protrusion, retraction and rotation, intramandibular rotation, saggital and forward rotation of the cheek region (suspensorium), gill cover rotation as a proxy for opercular linkage displacement and cranial elevation.

**Figure 3:**
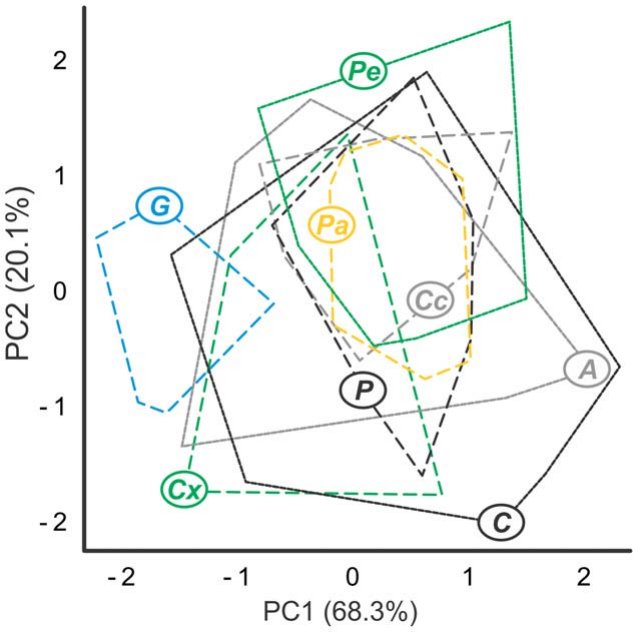
Limited diversity in angelfish feeding apparatus kinematics. Biplot of PC scores from the first two axes generated by a PCA on the kinematic dataset. Note the extensive centroid overlap for all taxa except the planktivore *Genicanthus* (blue). For other taxon labelling, see Table 1.

In this way, a total of 32 kinematics variables were sampled from high-speed video of each feeding event with kinematics means based on all bites from all individuals of a given species. Angular excursion measurements were left untransformed and linear measurements were corrected for individual head length. Excursion velocity variables were corrected for individual head length and log-transformed, while duration and timing variables were transformed into duty-factors using the total bite duration (***t***_TOT_) and then log-transformed. Variables were omitted from analysis only if there was significant auto-correlation between variables. The choice of which of two auto-correlated variables to remove was guided by biomechanical evaluations. This resulted in a final dataset of 23 informative variables that was subjected to further analyses. We verified that the resulting dataset did not violate assumptions for parametric analyses using the data normality examination in Systat v. 12. A MANOVA was used to evaluate the extent of variation in the dataset, and we then ran a principal component analysis (PCA) on the correlation matrix of the kinematic dataset (Figure 3).

**Figure 4:**
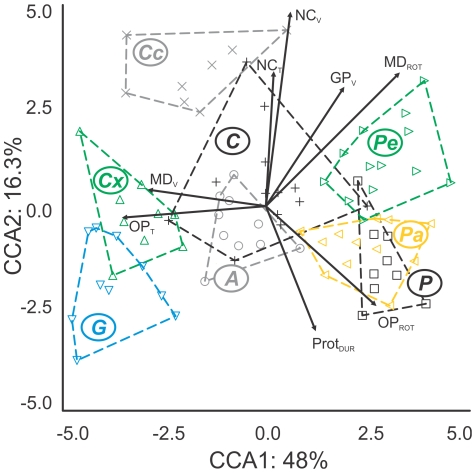
Influence of kinematics and body size on shaping the angelfish grab-and-tearing feeding mode. Scatter-plot of mean canonical scores for the first two axes generated by a DFA. Vectors for canonical loadings of informative kinematics variables (std. coeff. >0.59), indicate their species-dispersing effect across kinematic space. Body-size, increasing from left along CCA1, was a trait that was resistant to data transformations (see text). For taxon codes, see Table 1.

The PCA did not convincingly separate taxa across available multivariate kinematic space (see below). Therefore, a discriminate function analysis (DFA) was used to examine the extent to which kinematic variables could segregate taxa across available 2D-kinematic functional space (Statistica v.6.0). The canonical correspondence component of DFA is highly sensitive to subtle variation, and thus a powerful method when aiming to identify and maximize the display of variation among predefined groups [38]. To examine inter-specific differences in biting kinematics the canonical correspondence axes were tested for statistical significance using a nested ANOVA design with species as a fixed effect and individuals nested within species as random effect. F-ratios for the main effect of species were tested using the error mean square of individuals nested within species as the denominator [39]. Hypothesis testing followed by Bonferroni-corrected pair-wise comparisons of the least-square means (post-hoc tests) identified which taxa, if any, that differed across the significant axes of variation. Canonical Discriminate factors (Table 2) loading heavily along informative CCAs were visualised as scaled eigenvectors (Figure 4) to graphically illustrate their role in taxon segregation.

**Table 2:** Summary of canonical correspondence axes (CCA) and standardized coefficients (Std. Coeff.) of canonical variables obtained from the Discriminant Function Analysis (DFA) on angelfish feeding kinematic data.

	CCA	1	2	3
	Eigenvalue	8.00	2.71	2.29
	Variance explained	48%	16%	14%
Canonical variables		Std. Coeff.		
Timing (from *t*_0_)	**Opercular rotation**	**−.77**	−.03	.08
	Lower jaw depression	.34	.31	−.40
	Suspensorial rotation	.34	.19	.23
	**Gape expansion**	.29	.43	**.56**
	**Cranial elevation**	.15	**.69**	**.69**
	IMJ rotation	−.04	−.01	−.47
	**Jaw protrusion duration**	.20	**−.58**	**−.67**
	Jaw retraction duration	−.33	−.19	.17
Magnitude	**Opercular rotation**	**.57**	−.46	.14
	**Lower jaw rotation**	**.62**	**.55**	.08
	Suspensorial rotation	.46	.24	.17
	Gape expansion	−.02	.18	−.14
	Cranial elevation	−.43	−.37	−.29
	IMJ rotation	−.29	−.37	−.27
	Jaw protrusion	.17	.44	−.49
Velocity	**Opercular rotation**	−.42	−.23	**−.81**
	**Lower jaw rotation**	**−.61**	.09	−.19
	Suspensorial rotation	−.26	−.03	−.04
	**Gape expansion**	.43	**.60**	**.54**
	**Cranial elevation**	.18	**.99**	−.06
	IMJ rotation	−.38	.44	−.21
	Jaw protrusion	.29	−.06	.42
	Jaw retraction	.44	−.49	−.16

Strongly loading coefficients are boldfaced. If they load strongly along the two first canonical axes they are vector-plotted in Figure 4.

**Figure 5:**
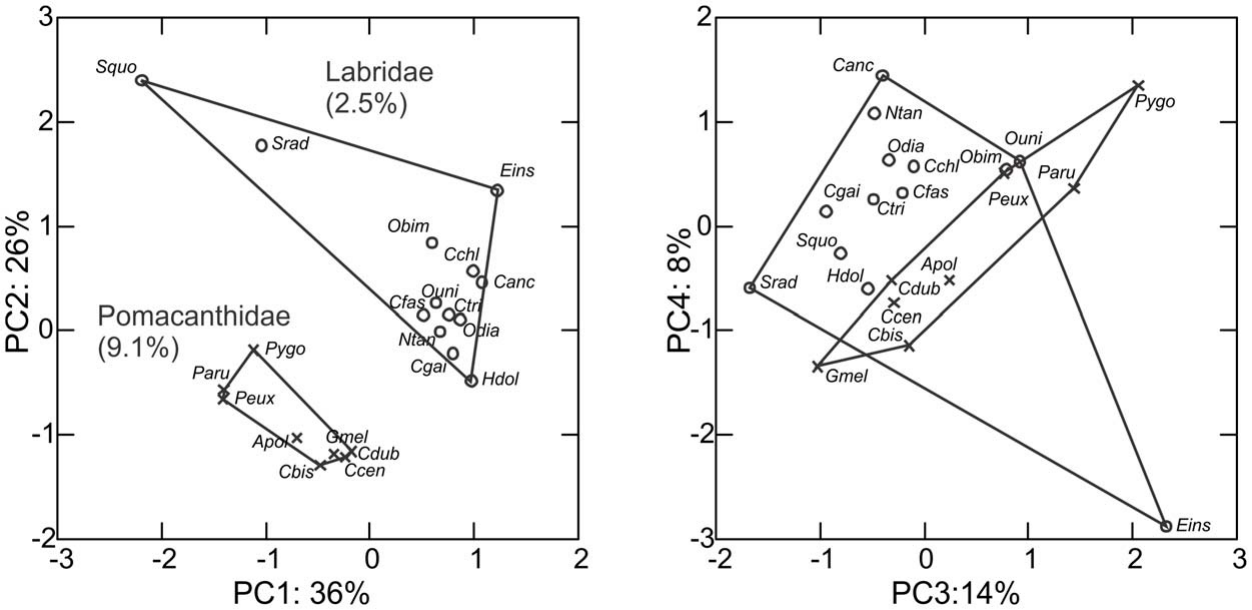
Low kinematic disparity in angelfish (f. Pomacanthidae) compared with wrasses and parrotfishes (f. Labridae). Biplots of the four first axes from a PCA on shared kinematics traits in pomacanthids and labrids. 2.5% of the nominal labrid species were sampled and they occupied twice the multivariate kinematics space of an almost four-fold denser sampling of angelfishes (9.1%). Literature origins of labrid data are given in Table S1. Taxon key: Ccen, *Centropyge bicolor*; Apol, *Apolemichthyes trimaculatus*; Cbis, *Centropyge bispinosa*; Pygo, *Pygoplites diacanthus*; Cdub, *Chaetodontoplus duboulayi*; Peux, *Pomacanthus sexstriatus*; Paru, *Pomacanthus semicirculatus*; Canc, *Choerodon anchorango*; Cgai, *Coris gaimard*; Hdol, *Hologymnus doliatus*; Ntan, *Novaculichthys taeniourus*; Odia, *Oxychelinus diagrammus*; Srad, *Sparisoma radians*; Squo, *Scarus quoyi*; Cchl, *Chelinus clorurus*; Cfas, *Chelinus fasciatus*; Ctri, *Chelinus trilobatus*; Obim, *Oxychelinus bimaculatus*; Ouni, *Oxychelinus unifasciatus*; Eins, *Epibulus insidiator*.

Functional disparity is defined as the range of diversity in a clade [40]. In order to investigate if the kinematic disparity of the angelfishes sampled here (9.1% of the nominal species) is high or low, we generated a metanalysis of published kinematics data for ram, suction and biting feeding in 13 labrid taxa (11 wrasses and two parrotfishes; representing 2.5% of the nominal species. The data overlap comprised 16 of the variables analysed for the angelfishes (Table S1), and included excursion amplitude, peak-timing and duration measures for lower jaw depression, jaw protrusion and retraction, gape expansion and occlusion, gill-cover rotation and cranial elevation (Figure S1). For the purpose of parametric analyses, missing data were substituted with median-values from all con-familiars. This method prevents rogue contribution to the disparity indices whilst retaining informative data from the affected taxa in the subsequent factor analyses [4]. Data were log-transformed as reported above. The resulting dataset was subjected to a principal component analysis on the correlation matrix, which we ran unconstrained, i.e. without designation of minimum Eigenvalues or maximum number of PC axes (Figure 5). We calculated the relative variance for each family from the PC factor score of each retrieved axis, scaled these variance-results to variance explained by each PC axis, and summed the taxon-specific variance across all axes, resulting in a parametric estimate of prey-capture kinematics disparity [4], [40].

## Results

### Functional morphology of the angelfish feeding apparatus

All angelfishes have heads that are laterally compressed, with cheek regions (suspensoria) that are reduced anteriorly, and oral jaws that can protrude far in front of the face because of their flexible and loose suspension (Figure 1). Major structural modifications, seen in all species, include: 1) an extra—intramandibular—joint in the lower jaw, between the dentary and articular bones of the mandible (Figure 1B, pt. 8), 2) significant flexion between the suspensorium (cheek region) and the neurocranium at a novel joint between the hyomandible and sphenoid (Figure 1B, pt. 5), and 3) a loosened anterior association of the cheek region with the neurocranium at the palatoquadrate and anterior pterygoid series. The latter two regions of flexion enable the cheek region to move anteriorly, relative to the neurocranium, which in return facilitates protrusion of the lower jaw – an exceptionally rare trait among modern bony fishes [25] (see Video S1, S2, S3, S4, S5, S6, S7, S8).

The lower jaw of most angelfishes rests at a characteristic acute dorsal incline from where the jaw protrudes directly forward (Figure 1). The only exception is seen in *Centropyge [Xiphypops]*, where a more obliquely horizontal resting angle leads to a more downwards directed protrusion of the jaws (Figure 2). Another structural novelty in these pygmy angelfishes is their flat frontal tooth margin. This is a shared trait between *Centropyge [Xiphypops]* and *Genicanthus* that contrasts with the arched margin seen in all other species. When these jaw morphology traits are optimized to the phylogeny of angelfishes (Figure 2) the restricted amount of morphological novelty is entirely confined to pygmy angelfishes; a recently evolved, small-bodied, heavily hybridizing and species-rich clade [32], [36], [41], [42], [43]. On the other hand, mapping of gut morphology traits onto the phylogeny (Figure 2) separates angelfishes into six groups that correspond strongly with trophic niche predictions based on field observations (e.g. [26]). Interestingly, herbivory has evolved twice, in some of the largest taxa as well as in a sub-genus of pygmy angelfishes.

### Diversity in feeding kinematics

All bites from all individuals are characterised by a slow phase of jaw protrusion (0.07–0.21 m s^−1^) with highly variable duration (0.054–0.3 s) leading to a maximum protrusion averaging 23% head length (HL), but only reaching 14% HL in the planktivore *Genicanthus*. Following maximum protrusion, a distinct and rapid jaw closure around the food item (0.012–0.059 s) is facilitated by rotation at the intramandibular joint. The jaw closure designates the time of bite (*t*_B_). Almost immediately following the time of bite, angelfishes tear off the food that is captured between their bristle-like teeth using a rapid jaw retraction (0.2–0.9 m s^−1^). The retraction of the jaws is augmented by a sideways head-jerk and pectoral-fin propelled rearward movement of the fish.

A MANOVA on the transformed kinematics variables indicated significant variance in the dataset (Wilk's lambda = 0.021, f_105_ = 3.36, p<0.001). A principal component analysis (PCA) on the correlation-matrix of the kinematics dataset returned two statistically informative PC axes (Figure 3), both with Eigenvalues over one, each of which explained 68.3% and 20.1% of the total amount of variance in the dataset. A MANOVA on the factor scores of these two PC axes revealed a significant species-effect on biting kinematics (Wilk's λ = 0.070; f1_4, 124_ = 24.538; p<0.001). However, hypothesis testing using pair-wise comparisons only recovered statistically significant differences between *Genicanthus* and all other taxa.

Given the lack of taxon-differentiation in the PCA, we ran a discriminant function analysis (DFA) on the same dataset, to determine if interspecific differences were too subtle to be recovered by the PCA. Seven canonical correspondence axes were returned of which the first three alone explained 78% of the dataset variance (Fig. 4; Table 2). Interspecific differences in biting kinematics were mainly driven by differences in timing and velocity variables (four of each category, see Table 2) whereas only two magnitude variables loaded heavily, both along CCA 1. Some variables loaded significantly along two axes, and among these were three descriptors feeding mechanisms that are ancestral to bony fishes. These included timing of cranial elevation (which aids in expanding the mouth), magnitude of lower jaw depression (which determines mouth expansion) and the velocity at which the mouth was opened. Only one descriptor of a feeding mechanism that is unique to angelfishes loaded heavily, namely the duration of lower jaw protrusion. Interestingly, none of the variables describing the movement of the angelfish functional innovations, namely rotation of the intramandibular or suspensorial joints, and the velocity of jaw retraction to tear off food, loaded significantly. Overall, comparable interspecific means and variances in biting kinematics were found. Separation of species along the three informative axes (table 2) described the function of the opercular linkage (CCA1), cranial elevation and jaw protrusion (CCA2) and gape expansion (CCA3). There was a clear influence of body-size along the first axis of this analysis (Figure 4), even though potentially sensitive variables had been body-size corrected prior to analyses.

### Feeding kinematics disparity

In the analysis comparing prey-capture functional disparity of angelfishes and labrids, four out of 16 PC axes explained more than 10% each of the total variance in the dataset, and these axes are shown in Figure 5. The disparity in prey-capture kinematics is doubled among the 2.5% of extant labrid species sampled randomly from the phylogeny, compared with 9.1% of the extant angelfish species sampled evenly across the phylogeny. Labrids have higher mean-values and exhibit more variability in their feeding kinematics than angelfishes for all variables except for angular excursions (Figure S1).

## Discussion

Among Indo-Pacific marine angelfishes, an unusual combination of structural and functional traits result in a novel and evolutionary conserved ‘grab-and-tearing’ feeding method. The morphology and kinematics underpinning this novel feeding method seems fundamentally different from other biters and from modern bony fishes in general. Comparisons with labrid reef fishes showed that the kinematic disparity of the grab-and-tearing feeding apparatus in angelfishes is restricted. The diverse trophic ecology of angelfishes appear better explained by gut morphological than by skull morphological disparity. However, given the descriptive nature of our skull morphological analysis, the notion of low structural disparity in the angelfish skull remains somewhat conjectural until a formal morphospace analysis is in place. Regardless, our kinematics and trophic ecological results reflect a contrasting trend in how morphology and kinematics underpin ecological patterns and the evolution of trophic diversity in aquatic feeding vertebrates.

### Structural and functional novelties may limit the kinematic disparity of biting

There was little variation seen in angelfish feeding apparatus morphology, and the function of the feeding apparatus varied little from a previous study of a generalised angelfish species [24]. All angelfishes have flexible connections between the cheek region and the neurocranium, permitting their jaws to protrude forward to an extreme degree. This novel trait enables them to reach food items that are might be inaccessible to most other biting taxa. Closure of mouth in protruded state serves to firmly grab food items between jaws that are armed with bristle-like teeth [22]. This grabbing of the food is facilitated by three novel joints, in particular the intramandibular joint (IMJ), which is an un-reversed angelfish synapomorphy, and clearly a key functional innovation [15]. Finally, a high-velocity jaw retraction serves to tear the food item from its attachment.

The few structural novelties are either synapomorphies relative to other bony fishes [24], [44], [45], or alternatively they arose once, as in the case of ventral jaw protrusion in pygmy angelfishes. The family lacks the repeated convergences on novel feeding mechanisms commonly seen in modern bony fishes, e.g. among the labrids [4], [46]. Loose suspensoria are rare among bony fishes and otherwise only seen in a few ram-suction feeding specialists [15], [25], [47], [48], [49]. Biters typically have very rigid cheek regions and therefore generally lack significant jaw protrusion [50], [51], [52]. Finally, the mouth-closing function of the IMJ in angelfishes contrasts with all other biting IMJ-bearers. Flexion at this joint in other groups serves to expand the gape to scrape more food area per bite [15], [24], [52], [53].

The few interspecific differences in angelfish feeding kinematics were far outweighed by much more subtle variation in the duration, onset-timing and velocity of kinematics that are ancestral to bony fishes. Interestingly, magnitude variables influenced species-segregation less than timing variables. The latter variables are under intrinsic behavioural control, and therefore likely to be less susceptible to morphological and phylogenetic constraints [54], [55], [56], [57].

A common idea is that structural novelty leads to functional decoupling of associated structures [58], [59], [60], in turn enhancing the potential for clade diversification [9]. It has been suggested that IMJs might channel a functional decoupling of the mandible by dividing it into two mechanical units, thereby increasing the complexity of the feeding apparatus [61]. Certainly, the unique gape-occluding IMJ function in angelfishes does represent an increased functional versatility associated with IMJ-bearing mandibles [15]. However, our data indicate a profound functional conservatism across angelfishes, clearly contradicting the notion of functional decoupling. In fact, the presence of an IMJ appears to pose significant constraints on the versatility of feeding apparatus function. This is best reflected by the only significant motion change across the family leading to a restricted flexion at the IMJ in the planktivorous *Genicanthus* compared with its obligate biting sister taxa [15].

### Radiation of angelfishes via negotiation of ecological thresholds

The ‘grab-and-tearing’ feeding system arose once and underwent a limited amount of change during the 38 million years of evolution in this ecologically successful family. How is the high level of ecological versatility, clearly reflected by gut morphology and field observations, explained by such stereotyped feeding morphology and biting kinematics? The unusual and highly versatile feeding apparatus, combined with the evolution of an order of magnitude variation in body size, appears to have permitted angelfishes to negotiate several ecological thresholds [62] formed by the challenges of feeding on their food of choice.

Our analyses of angelfish gut morphology substantiated earlier notions of herbivory having arisen independently in the large-bodied *Pomacanthus [Euxiphipops]* clade as well as in the pygmy angelfish subgenus *Centropyge [Xiphypops]* [26], [27], [28]. Transitions to other trophic niches also received good support, based on the evolution of muscular stomachs (gizzards) and hindgut fermentation chambers [63], [64], [65].

The high diversity in angelfish gut morphology combines with an overriding role of body size on the trophic evolution of the family. Benefits of being large are most obvious among spongivores and robust omnivores, where an associated increase in the forcefulness of grab-and-tearing facilitates the rupturing or dislodging of structurally resilient sponges and tunicates [24]. The same robust feeding mode also permits *P. [Euxiphipops]* to feed on sturdy calcareous or foliaceous algae [19].

Pygmy angelfishes show the only major morphological divergence within the family. In *Centropyge [Xiphypops]*, the physical force-production constraints inherent to a small body size have led to gracile combing or shearing strategies and therefore feeding on delicate foliaceous algae. Their oblique jaw protrusion means that the body can remain parallel with the substratum during feeding, which likely improves the predator-avoidance response [66], [67]. Thus, herbivorous pygmy angelfishes are able to venture away from shelter and feed on epilithic algae that occupy exposed sunlit substrata [68]. In contrast, *Centropyge [Centropyge]* feed on attached colonial invertebrates close to shelter or tucked away within the reef-matrix [69]. Their terminal jaw protrusion dictates an acute body orientation relative to the substratum during feeding, which likely carries less of a predation risk due to their sheltered foraging sites.

The clear differences in jaw structure and function, and in relative gut length, between the two *Centropyge* clades, support the elevation of *C. [Xiphypops]* to full generic status. The clade of herbivorous, small-bodied and heavily hybridizing *Centropyge [Xiphypops]* and their planktivorous sister-taxon *Genicanthus* recently underwent pronounced and rapid speciation. Together, they comprise 25% of all angelfishes. Interestingly, this could be a product of increased disparity as a result of hybridization [10], [43].

*Genicanthus* differed from all other angelfishes by having a reduced degree of motion in most of its ancestral feeding mechanisms. Restricted movements of its diminutive mouth, coupled with the shortest and least differentiated gin the family corresponds with a secondary functional reversal to planktivory [23], [31], [65], [70]. Reversals to the ancestral suction feeding mode [44], [45] are likely worth future in-depth exploration.

### Functional innovations, disparity and many-to-one mapping in macroevolution

Our study is one of the first to quantify the kinematics underpinning the biting feeding mode, and the first to sample kinematics in a systematic manner across a monophyletic clade mainly comprised of biters. The kinematics disparity characterizing angelfish biting was only half of that seen in labrid wrasses and parrotfishes, which have repeatedly evolved biting as well as ram-suction feeding strategies. Our disparity measurements must be considered preliminary because of the small number of species sampled. Moreover, ancestry-calibrated comparative analyses were impossible, as no angelfish fossils are available. Regardless, two lines of inference support our disparity estimates: The uneven phylogenetic sampling of labrid feeding kinematics, and the substitution of missing data-points with median values (Table S1) likely renders a conservative measure of labrid functional disparity (Figure S1). Moreover, given the comparable clade ages (50 myo for Pomacanthidae, Figure 2. *vs.* Labridae, 60 myo; [71]) our results are not likely to be significantly altered by ancestry-calibrations.

A general lack of morphological change in angelfishes since the Eocene reflects the status of most reef fish families [72]. This pattern is only contrasted by a few groups, most notably the Labridae [46], [53]. In fact, labrids might be exceptional among high-diversity reef fish groups, in possessing considerable structural as well as functional disparity. In contrast, our data from a successful biting group show that a few structural novelties can prompt diversification along multiple ecological axes, without the evolution of functional disparity. This finding questions the general utility of many-to-one mapping theory in explaining general diversification processes [73]. Consequently, synthesis of kinematics data with morphological, biomechanical and ecological data, although more logistically demanding, remains the most promising way to improve our understanding of how speciose and successful assemblages evolve. By taking this approach, we have shown that novel morphological traits may indeed constrain functional versatility. We also cast angelfishes as an intriguing macroevolutionary example of how a successful group can evolve considerable trophic diversity although they possess low structural and functional disparity in their feeding apparatus.

## Supporting Information

Figure S1 **Summary of feeding kinematics in f. Pomacanthidae and f. Labridae.** Labrids have more variable kinematics, as indicated by standard deviation whiskers, and typically higher mean values than pomacanthids. Abbreviations: *Amplitudes*; MDDep, mandible depression; OPRot, opercular rotation; NCEle, neurocranial elevation; GPExp, gape expansion; JAWprot, jaw protrusion; *Maximum-timing*; MDDepTim, mandible depression; OPTim, opercular rotation; NCTim, neurocranial elevation; GPTim, gape expansion; PROTtim, jaw protrusion; *Durations*; MDdepDur, lower jaw depression; OPDur, opercular rotation; NCDur, neurocranial elevation; GPDur, gape expansion; GPCLDur, gape occlusion; PROTdur, jaw protrusion; RETdur, jaw retraction; BITEdur, total prey capture event. Data as listed in Table S1, but without median entries.
(TIF)

Table S1 **Uncorrected variables for comparative kinematics analyses.** Data used came from: ^a^Present study, ^b^ [38], ^c^Average of values reported in [38] and in [74], ^d^ [14], ^e^ [75], ^f^ [74], ^g^ [25]. Missing data were substituted with median value calculated using data from con-familiars (in boldface italics).
(DOC)

Video S1 ***Centropyge [Centropyge] bicolor*** **feeding on Ghost shrimp (** ***Acetes*** **).** The video was recorded at 200 fps to view 8 times slower than real-time.
(MPG)

Video S2 ***Apolemichthys trimaculatus*** **feeding on a sponge.** The video was recorded at 200 fps to view 8 times slower than real-time.
(MPG)

Video S3 ***Genicanthus melanospilos*** **feeding, first on attached- then a free-floating piece of squid (** ***Loligo*** **).** The video was recorded at 500 fps to view 20 times slower than real-time.
(MPG)

Video S4 ***Centropyge [Xiphypops] bispinosa*** **feeding on turf algae.** The video was recorded at 200 fps to view 8 times slower than real-time.
(MPG)

Video S5 ***Pygoplites diacanthus*** **feeding on a sponge.** The video was recorded at 200 fps to view 8 times slower than real-time.
(MPG)

Video S6 ***Chaetodontoplus duboulayi*** **feeding on a sponge.** The video was recorded at 200 fps to view 8 times slower than real-time.
(MPG)

Video S7 ***Pomacanthus [Euxiphipops] sexstriatus*** **feeding on a sponge.** The video was recorded at 200 fps to view 8 times slower than real-time.
(MPG)

Video S8 ***Pomacanthus [Arusetta] semicirculatus*** **feeding on a sponge.** The video was recorded at 200 fps to view 8 times slower than real-time.
(MPG)
