# Supplementary material for: Evolution of High Trophic Diversity Based on Limited Functional Disparity in the Feeding Apparatus of Marine Angelfishes (f. Pomacanthidae)
Source: PLoS One. 2011 Sep 1;6(9):e24113. doi: 10.1371/journal.pone.0024113 (PMC3164712; doi:10.1371/journal.pone.0024113)
Supplement: Table S1 — Uncorrected variables for comparative kinematics analyses. Data used came from: aPresent study, b [38], cAverage of values reported in [38] and in [74], d [14], e [75], f [74], g [25]. Missing data were substituted with median value calculated using data from con-familiars (in boldface italics). (DOC) [file pone.0024113.s002.doc]

**Table S1.** Uncorrected taxon variables for comparative kinematics analyses.

| A. Amplitude | Mandible depression angle (°) | opercular rotation angle (°) | cranial elevation angle (°) | gape expansion (mm) | jaw protrusion (mm) |
| --- | --- | --- | --- | --- | --- |
| *Centropyge bicolor^a^* | 49.8 | 4.8 | 7.6 | 4.0 | 5.8 |
| *Apolemichthyes trimaculatus^a^* | 46.9 | 5.8 | 7.2 | 6.2 | 8.1 |
| *Genicanthus melanospilos^a^* | 27.3 | 3.3 | 3.0 | 3.0 | 3.3 |
| *Centropyge bispinosa^a^* | 40.0 | 5.3 | 6.9 | 2.5 | 4.3 |
| *Pygoplites diacanthus^a^* | 47.1 | 12.4 | 10.8 | 7.8 | 9.0 |
| *Chaetodontoplus duboulayi^a^* | 47.7 | 6.5 | 7.0 | 8.6 | 8.9 |
| *Pomacanthus sexstriatus^a^* | 50.4 | 6.2 | 5.1 | 13.5 | 15.0 |
| *Pomacanthus semicirulatus^a^* | 49.8 | 9.4 | 8.1 | 10.7 | 14.5 |
| *Choerodon anchorango^b^* | 26.2 | ***8.0*** | 4.1 | 15.5 | 2.5 |
| *Coris gaimard^b^* | 24.1 | ***8.0*** | 4.1 | 8.6 | 2.2 |
| *Hologymnus doliatus^b^* | 16.2 | ***8.0*** | 9.9 | 6.8 | 1.6 |
| *Novaculichthys taeniourus^b^* | 42.0 | ***8.0*** | 4.9 | 12.7 | 2.2 |
| *Oxychelinus diagrammus^c^* | 30.0 | 7.5 | 6.7 | 13.8 | 4.5 |
| *Sparisoma radians^d^* | 32.0 | ***8.0*** | 0.7 | 12.0 | 1.9 |
| *Scarus quoyi^d^* | 32.0 | ***8.0*** | 1.2 | 8.7 | 3.7 |
| *Chelinus chlorurus^e^* | 40.0 | 7.0 | 7.0 | 14.0 | 5.0 |
| *Chelinus fasciatus^f^* | 32.2 | 8.2 | 5.3 | 12.0 | 5.0 |
| *Chelinus trilobatus^f^* | 30.5 | 7.6 | 5.9 | 12.0 | 5.0 |
| *Oxychelinus bimaculatus^f^* | 33.2 | 6.9 | 9.2 | 7.0 | 3.0 |
| *Oxychelinus unifasciatus^f^* | 43.7 | 7.5 | 12.6 | 15.0 | 6.0 |
| *Epibulus insidiator^g^* | 5.0 | 10.5 | 11.0 | 11.0 | 32.0 |

| B. Timing (ms from ***t***_0_) | Mandible depression | opercular rotation | cranial elevation | gape expansion | Jaw protrusion |
| --- | --- | --- | --- | --- | --- |
| *Centropyge bicolor^a^* | 0.4 | -2.2 | 0.9 | -12.0 | -2.2 |
| *Apolemichthyes trimaculatus^a^* | 0.0 | -11.0 | -8.0 | -19.9 | -6.9 |
| *Genicanthus melanospilos^a^* | 0.2 | -12.8 | 0.9 | -18.8 | -5.9 |
| *Centropyge bispinosa^a^* | -7.8 | -8.9 | -6.7 | -15.6 | -8.9 |
| *Pygoplites diacanthus^a^* | -6.0 | -4.6 | -5.6 | -32.2 | -10.0 |
| *Chaetodontoplus duboulayi^a^* | -2.7 | -7.3 | -3.5 | -19.6 | -10.0 |
| *Pomacanthus sexstriatus^a^* | -10.0 | -28.5 | -10.8 | -63.5 | -20.8 |
| *Pomacanthus semicirulatus^a^* | -10.0 | -14.2 | -13.2 | -48.8 | -25.0 |
| *Choerodon anchorango^b^* | 100.7 | ***19.0*** | 123.3 | ***31.0*** | 123.1 |
| *Coris gaimard^b^* | 54.6 | ***19.0*** | 58.6 | ***31.0*** | 52.0 |
| *Hologymnus doliatus^b^* | 38.3 | ***19.0*** | 56.7 | ***31.0*** | 32.6 |
| *Novaculichthys taeniourus^b^* | 38.7 | ***19.0*** | 42.9 | ***31.0*** | 44.3 |
| *Oxychelinus diagrammus^c^* | 40.5 | 20.0 | 36.2 | 35.0 | 50.3 |
| *Sparisoma radians^d^* | ***41.0*** | ***19.0*** | -66.7 | 80.7 | -137.0 |
| *Scarus quoyi^d^* | ***41.0*** | ***19.0*** | -174.0 | -42.5 | -48.0 |
| *Chelinus chlorurus^e^* | 40.0 | 30.0 | 40.0 | 40.0 | 30.0 |
| *Chelinus fasciatus^f^* | 30.0 | 20.0 | 35.0 | 35.0 | 35.0 |
| *Chelinus trilobatus^f^* | 25.0 | 20.0 | 25.0 | 25.0 | 25.0 |
| *Oxychelinus bimaculatus^f^* | 20.0 | 10.0 | 20.0 | 20.0 | 20.0 |
| *Oxychelinus unifasciatus^f^* | 20.0 | 10.0 | 20.0 | 20.0 | 20.0 |
| *Epibulus insidiator^g^* | ***41.0*** | 20.0 | 30.0 | 40.0 | 20.0 |

| C. Duration (ms) | mandible depression | opercular rotation | cranial elevation | gape expansion | jaw protrusion | Jaw retraction |
| --- | --- | --- | --- | --- | --- | --- |
| *Centropyge bicolor^a^* | 37.3 | 44.0 | 41.3 | 37.8 | 54.2 | 10.7 |
| *Apolemichthyes trimaculatus^a^* | 65.2 | 85.5 | 74.2 | 71.5 | 113.9 | 14.4 |
| *Genicanthus melanospilos^a^* | 54.0 | 56.7 | 46.8 | 48.2 | 64.0 | 20.3 |
| *Centropyge bispinosa^a^* | 36.7 | 47.2 | 35.4 | 32.8 | 61.7 | 15.6 |
| *Pygoplites diacanthus^a^* | 144.6 | 72.1 | 95.0 | 126.1 | 157.1 | 12.2 |
| *Chaetodontoplus duboulayi^a^* | 76.3 | 78.2 | 78.5 | 62.4 | 94.9 | 14.7 |
| *Pomacanthus sexstriatus^a^* | 246.9 | 185.4 | 249.2 | 201.1 | 274.2 | 30.4 |
| *Pomacanthus semicirulatus^a^* | 194.8 | 246.3 | 272.7 | 222.5 | 300.1 | 21.7 |
| *Choerodon anchorango^b^* | 120.0 | ***49.0*** | ***54.0*** | ***45.0*** | 123.1 | 73.0 |
| *Coris gaimard^b^* | 90.0 | ***49.0*** | ***54.0*** | ***45.0*** | 52.0 | 35.3 |
| *Hologymnus doliatus^b^* | 65.0 | ***49.0*** | ***54.0*** | ***45.0*** | 32.6 | 29.4 |
| *Novaculichthys taeniourus^b^* | 75.0 | ***49.0*** | ***54.0*** | ***45.0*** | 44.3 | 34.0 |
| *Oxychelinus diagrammus^c^* | 75.0 | 45.0 | 60.0 | 35.0 | 50.3 | 29.7 |
| *Sparisoma radians^d^* | ***69.0*** | ***49.0*** | 232.0 | 208.0 | 176.0 | ***40.0*** |
| *Scarus quoyi^d^* | ***69.0*** | ***49.0*** | 159.0 | 204.0 | 147.0 | ***40.0*** |
| *Chelinus chlorurus^e^* | 60.0 | 50.0 | 65.0 | 40.0 | 30.0 | 60.0 |
| *Chelinus fasciatus^f^* | 65.0 | 65.0 | 65.0 | 65.0 | 65.0 | ***40.0*** |
| *Chelinus trilobatus^f^* | 45.0 | 40.0 | 45.0 | 45.0 | 45.0 | 20.0 |
| *Oxychelinus bimaculatus^f^* | 40.0 | 35.0 | 40.0 | 40.0 | 40.0 | ***40.0*** |
| *Oxychelinus unifasciatus^f^* | 50.0 | 50.0 | 50.0 | 50.0 | 50.0 | ***40.0*** |
| *Epibulus insidiator^g^* | ***69.0*** | 60.0 | 110.0 | 40.0 | 34.7 | 111.3 |

^a^Present study, ^b^[38], ^c^Average of values reported in [38] and in [74], ^d^[14], ^e^[75], ^f^[74], ^g^ [25]. Missing data were substituted with median value calculated from data on con-familiars (in boldface italics).
